# The intrahemispheric functional properties of the developing sensorimotor cortex are influenced by maturation

**DOI:** 10.3389/fnhum.2015.00039

**Published:** 2015-02-17

**Authors:** Marika Berchicci, Gabriella Tamburro, Silvia Comani

**Affiliations:** ^1^BIND - Behavioral Imaging and Neural Dynamics Center, University “G. d'Annunzio” of Chieti-PescaraChieti, Italy; ^2^Department of Movement, Human and Health Sciences, University of Rome “Foro Italico,”Rome, Italy; ^3^Department of Medicine and Aging Sciences, University “G. d'Annunzio” of Chieti-PescaraChieti, Italy; ^4^Department of Neuroscience, Imaging and Clinical Sciences, University “G. d'Annunzio” of Chieti-PescaraChieti, Italy; ^5^Casa di Cura Privata Villa SerenaCittà Sant'Angelo, Italy

**Keywords:** sensorimotor cortex, functional organization, connectivity, synchronization likelihood, segregation, integration, rolandic mu rhythm

## Abstract

The investigation of the functional changes in the sensorimotor cortex has important clinical implications as deviations from normal development can anticipate developmental disorders. The functional properties of the sensorimotor cortex can be characterized through the rolandic mu rhythm, already present during infancy. However, how the sensorimotor network develops from early infancy to adulthood, and how sensorimotor processing contributes to the generation of perceptual-motor coupling remains largely unknown. Here, we analyzed magnetoencephalographic (MEG) data recorded in two groups of infants (11–24 and 26–47 weeks), two groups of children (24–34 and 36–60 months), and a control group of adults (20–39 years), during intermixed conditions of rest and prehension. The MEG sensor array was positioned over the sensorimotor cortex of the contralateral hemisphere. We characterized functional connectivity and topological properties of the sensorimotor network across ages and conditions through synchronization likelihood and segregation/integration measures in an individual mu rhythm frequency range. All functional measures remained almost unchanged during the first year of life, whereas they varied afterwards through childhood to reach adult values, demonstrating an increase of both segregation and integration properties. With age, the sensorimotor network evolved from a more random (infants) to a “small-world” organization (children and adults), more efficient both locally and globally. These findings are in line with prior studies on structural and functional brain development in infants, children and adults. We could not demonstrate any significant change in the functional properties of the sensorimotor cortex in the prehension condition with respect to rest. Our results support the view that, since early infancy, the functional properties of the developing sensorimotor cortex are modulated by maturation.

## Introduction

In the cerebral cortex, functional domains such as visual, cognitive or sensorimotor control rely on the development of distinct and interconnected cortical and subcortical regions (Tau and Peterson, 2009). For instance, the transient loss of face orientation discrimination at 2 months of age may result from a conflict between subcortical and cortical pathways, also known as *transient functional deterioration* (Nakano and Nakatani, 2014). The subsequent recovery by 4–6 months of age can be interpreted as an establishment of coordination between the two systems. U-shaped changes in a given behavior have been observed in reaching (Butterworth, 1989) and cross-modal orientation (Taga et al., 2002). The developmental organization of these circuits is a complex process that begins at early gestational age (Kostovic et al., 1995) and continues until adulthood. The brain development begins with neuronal proliferation and proceeds with migration, apoptosis, synaptogenesis, pruning, myelination and cortical thinning (Giedd et al., 2009). These events are temporally overlapped and are genetically determined, epigenetically directed and environmentally influenced (Tau and Peterson, 2009) by sensorimotor and cognitive experiences.

Investigators working in the pediatric field have been particularly interested in the developmental properties of the mu rhythm, which reflects sensorimotor processing in the fronto-parietal network (Pineda, 2005). The adult mu rhythm, which falls within the alpha frequency range (8–13 Hz), is strongly inhibited (i.e., desynchronized, suppressed) before and during the execution of a bodily movement (Cheyne et al., 2014), during the observation (Jarvelainen et al., 2004; Vogt et al., 2007) and the imagination (Grafton et al., 1996; Molnar-Szakacs et al., 2006) of a goal-directed action, and also during sensorimotor stimulation (Cheyne et al., 2003). The mu rhythm modulation is considered a neurophysiological measure of the mirror neuron system (MNS), which is a neuronal mechanism that matches perception and action, allowing goal-directed action understanding (Hari et al., 2000; Hummel et al., 2002; Muthukumaraswamy and Johnson, 2004; Depretto et al., 2006).

A growing body of literature indicates that action experience may also modulate the mu rhythm desynchronization (Calvo-Merino et al., 2006; van Elk et al., 2008; Cannon et al., 2013; Ruther et al., 2014). Recent work on the infant mu rhythm indicates that it is present in infancy (Stroganova et al., 1999; Marshall et al., 2002; Berchicci et al., 2011; Marshall and Meltzoff, 2011), although its peak activity occurs at lower frequency ranges as compared to older children and adults, as it occurs for other brain rhythms, such as the theta and delta rhythms (Orekhova et al., 2006; Cuevas et al., 2014). It has been observed that the infant mu rhythm desynchronizes during the execution of a goal-directed action as well as the observation of a previously learnt action, and that infants' self-experience has an influence on the expectations about others action. These findings support the notion that the action-perception coupling network appears early in life (Marshall and Meltzoff, 2014). However, no general agreement exists on the mechanisms leading to the onset of this network. One of the most popular views claims that perceptual-motor coupling is present at birth and is merely shaped by experience (Lepage and Theoret, 2007; Simpson et al., 2014). An alternative position suggests that sensorimotor experience plays a critical role in the generation of perceptual-motor coupling through general associative learning processes (Heyes, 2001; Cook et al., 2014). Support to this second view comes from studies (Southgate et al., 2009; de Klerk et al., 2014) where the authors showed how visual experience alone increased sensorimotor cortex engagement by activating the previously established visuomotor associations (Greenough et al., 1987; Stiles and Jernigan, 2010).

Indeed, the postnatal period represents a time of dramatic change in the brain structure and function. The brain grows to about 70% of its adult size by 1 year of age, to about 80% by age 2, and to 90% of its adult size between the age of 2 and 5 years, which is known as the “plateau” phase of development (Knickmeyer et al., 2008). With time, local connections within cortical circuits, especially in sensorimotor and visual cortices (Fransson et al., 2007), are fine-tuned, and long-range connections among circuits produce an increasingly unified and functionally organized neural network. Sets of regions that share temporally correlated activity are believed to represent and become functional networks (Damoiseaux et al., 2006), and some studies had provided advanced understanding of the functional architecture of the human brain during early development (Gao et al., 2009; Doria et al., 2010; Dosenbach et al., 2010).

Functional connectivity magnetic resonance imaging (fcMRI) has proved useful in newborn studies. Indeed, it offers insight into the earliest forms of cerebral connectivity in very young infants (Power et al., 2010; Smyser et al., 2011). Damaraju et al. (2014) recently characterized the development of intrinsic connectivity networks (ICNs) in infants aged between 4 and 9 months with resting state MRI (rsMRI) performed while sleeping without sedative medication. They observed that, with age, the connectivity strength decreased within local networks and increased between more distant networks. Other researchers (Gilmore et al., 2011) have shown that, from birth to 2 years of age, cortical thinning proceeds in a back to front direction and occurs first in the sensorimotor areas, followed by association areas and lastly by higher-order cortical areas, such as the prefrontal cortex and the posterior parietal cortex. Two types of age-related changes (from childhood to adulthood) in functional connectivity have been described so far: decreases in local connectivity among anatomically adjacent, but functionally distinct brain regions, because they are integrated in their own brain networks; increases in long-range connectivity among nodes that comprise each network (Fair et al., 2007; Kelly et al., 2009).

In contrast to the high spatial resolution but low temporal resolution of rsMRI and fcMRI, magnetoencephalography (MEG) and electroencephalography (EEG) enable the measurement of functional connectivity with high temporal resolution and medium level spatial resolution. Few studies (Ellingson, 1964; Vanhatalo and Kaile, 2006) have demonstrated the evolution of electro-cortical activity in the infant brain by means of EEG recording, with regional variability and increasingly synchronous activity between bilateral homologous regions. Event related potential (ERP) findings suggested that sensorimotor networks undergo rapid development during the first year of life (Bell and Fox, 1992; Lin et al., 2008). To estimate cortical functioning, Bell and Wolfe (2007) examined the developmental changes of electrical activity during a working memory task in infants and children by means of EEG power and coherence (a spectral measure of the functional coupling between neural generators), and observed similar changes in both measures: the widespread brain electrical activity typical in infancy (8 months of age) became more localized during early childhood (3 years of age). Righi et al. (2014) conducted a longitudinal study in infants at 6 and 12 months of age, and found that infants at risk of autism spectrum disorder had lower functional connectivity between frontal and parietal regions as indexed by linear coherence in gamma frequency band. Other very recent studies (Keehn et al., 2013; Imai et al., 2014) employed the near-infrared spectroscopy (NIRS) to look at the functional connectivity in term, pre-term, and Down syndrome infants, observing increased longer distance functional connectivity over the first year of life in normal developing infants as compared to pathological infants.

MEG was demonstrated to be a suitable method to investigate the function of the developing brain during infancy and childhood due to its non-invasive nature, its excellent temporal resolution and, with recent devices, also good spatial resolution. A technical and practical advantage of MEG systems in pediatric applications is that MEG signals are unaffected by the immature skull features such as fontanels, allowing for longitudinal neuro-developmental studies. In a recent study employing MEG recordings over the contra-lateral hemisphere during a prehension task and power spectrum analysis, Berchicci et al. (2011) reported the presence of idling mu rhythm at rest and its suppression during prehension in infants (from 11 to 47 weeks of age) and pre-school children (from 2 to 5 years). In particular, they showed that mu rhythm peak frequency increases as a function of age (from 2.75 Hz at 11 weeks of age to 9.5 Hz at 3 years of age), and undergoes a rapid maturation during the first year of life. Based on the time-frequency analysis performed, the specific MEG signal waveform, and the position of the cluster of channels showing maximum mu rhythm desynchronization during prehension over the subject's head, Berchicci et al. (2011) also suggested a sensorimotor generator site for mu oscillatory activity.

Few studies using diffusion tensor imaging based fiber tractography or resting state fMRI have adopted a graph theoretical approach to characterize the developmental properties of the child brain. These studies have focused on the assessment of structural brain maturation (Lebel et al., 2008), on the analysis of intrinsic functional connectivity (Supekar et al., 2009), on the evolution of brain connectivity patterns (Yap et al., 2011), and on the development of neural systems underlying cognition (Fair et al., 2009). In general, they showed that the child brain develops with maturation from strong local connectivity toward a more distributed, predominantly functional based, connectivity pattern characterized by stronger integration.

However, little information is provided on the developmental trajectories of the functional properties of the sensorimotor cortex, and on how sensorimotor processing is modulated by sensorimotor experience. Here we analyzed the MEG data collected in our previous study (Berchicci et al., 2011) using a measure of functional connectivity and graph theoretical concepts to contribute to the understanding of the functional organization of the developing sensorimotor cortex from early infancy to adulthood. In an attempt to verify whether sensorimotor processing contributes to the generation of perceptual-motor coupling, we analyzed the MEG data recorded in two experimental conditions: at rest and during the execution of a prehension task.

We typified the functional organization over the sensorimotor cortex by means of functional connectivity and efficiency measures. Since the developmental properties of the mu rhythm reflect sensorimotor processing (Pineda, 2005), all measures were calculated within an individual mu rhythm frequency band to ensure that all findings referred to the sensorimotor network. Synchronization likelihood (Stam and van Djik, 2002), used in brain studies to quantify the probability for the functional interdependencies between neural signals, was used to reconstruct functional connectivity maps over the sensorimotor cortex. The type of functional efficiency over the sensorimotor network was estimated by means of segregation and integration measures (Stam, 2000) calculated on the functional connectivity maps. Functional segregation measures (i.e., mean clustering coefficient and local efficiency) quantify the specialized information processing occurring within densely interconnected groups of brain regions (Rubinov and Sporns, 2010), whereas functional integration measures (i.e., characteristic path length, Watts and Strogatz, 1998 and global efficiency, Latora and Marchiori, 2001) reckon the ability of a functional network to rapidly combine specialized information processing from distributed brain regions.

We also tried to explore whether any differences could be observed in the functional properties of the sensorimotor cortex during the execution of a prehension task, in order to provide information useful to understand the contribution of sensorimotor processing to the development of perceptual-motor coupling in infants and children. To this aim, we compared the global measures of functional connectivity and efficiency over the sensorimotor cortex at rest with those related to the execution of a prehension task. Prehension is one of the most remarkable examples of perception and action coupling in infants, since reaching for an object is guided by perceptual information on the relation between the self and the environment, which continuously changes as the posture (e.g., the motor system) is adjusted to that information (Hatwell, 1987). Therefore, prehension is a motor task suitable to explore whether sensorimotor processing contributes to the generation of perceptual-motor coupling.

Our investigation adds to prior studies that have considered either the rest condition or the observation of actions (Gilmore et al., 2011; Virji-Babul et al., 2012; Damaraju et al., 2014; Rotem-Kohavi et al., 2014), since we studied the functional properties over the developing sensorimotor cortex not only at rest but also during prehension. Moreover, by including infants below 6 months of age in our study population, we provided a valuable adjunct to studies on the developing brain.

Based on the knowledge that children and young-adults' brains have “small-world” organization at the global level (Supekar et al., 2009), and on the notion that fronto-temporal connections develop more slowly than other regions (Lebel et al., 2008), we expect that the functional properties over the sensorimotor network evolve, from infants to children and adults, toward a more efficient “small-world” organization. As well, based on the hypothesis that sensorimotor processing plays a role in the generation of perceptual-motor coupling, we expect to observe different functional organizations over the sensorimotor cortex for the rest and prehension conditions.

## Materials and methods

### Participants

Data collection was performed in infants, children and adults following a cross-sectional design. Participants were selected from a larger database of subjects (see Berchicci et al., 2011). Subjects experiencing any serious illnesses or developmental problems since birth (i.e., traumatic brain injury, seizures, and congenital conditions), or receiving any long-term medication were excluded from the study. Out of the 43 healthy infants enrolled (<12 months of age), 25 infants met all inclusion and exclusion criteria. Chronological age at entry in the study ranged between 11 and 47 weeks (mean = 24.9, *SD* = 10.8). The functional development of all infants was examined with the Kent Inventory of Developmental Skills (KIDS; Reuter et al., 1996). All infants were found to function within the normal range for age. Parent report was used to assess Apgar score (Apgar, 1953) at birth, which were all within normal limits (i.e., 8–10). However, due to poor signal quality that prevented a reliable calculation of the functional connectivity and efficiency measures, 11 infants had to be excluded from the study (see details in Section Statistical Analysis). Eighteen healthy children aged between 24 and 60 months were enrolled and met all inclusion and exclusion criteria, but, due to motion artifacts, only 12 children were included in the study population (mean = 37.3, *SD* = 12). Six right-handed adults (mean = 28.3, *SD* = 7.8) participated in the study as control group.

Based on the results of our prior study (see Berchicci et al., 2011), we knew that a large variation of the mu rhythm peak frequency occurred during the first year of life, indicating a rapid functional development. For this reason, we decided to split the group of infants in two age groups, one including the infants <6 months of age, and another including the infants <12 months of age. To have a similar number of subjects in all study groups, we split also the group of children in two age groups, one including the children with an age range of 24–34 months, and another including the children with an age range of 36–60 months. Details on the age groups can be found in Section Statistical Analysis.

The protocol was reviewed and approved by the Human Research Review Committee at the University of New Mexico Health Sciences Center and written informed consent was obtained from participant's guardians or adult participants after the description of the study protocol. Infants and children were recruited at day-care centers and from the community using word of mouth, brochures posted on campus, and social networks.

### Procedure and task

The experimental setup is only briefly described herein; details can be found in our previous study (see Berchicci et al., 2011). Neuromagnetic activity was recorded using a multi-channel pediatric magnetoencephalography (MEG) system (Okada et al., 2006) for hemispheric recordings. The sensor array operated in a magnetically shielded room, consisted of 76 first-order axial gradiometers, and had a headrest with a smooth outer surface made of thick fiberglass. Inter-sensor distance was approximately 13 mm center-to-center. The headrest was based on a standard reference for the head size of babies. Since the thickness of the neonatal scalp and skull is about 3–4 mm, brain activity could be measured a few millimeters above the brain surface, providing excellent sensitivity.

Since participants performed the assigned tasks with their right hand, they were positioned on the MEG bed on their left side, and a pillow was used to support their back, if necessary. The left hemisphere of the head was positioned on the MEG headrest in order to cover the sensorimotor areas. To ensure child safety and to promote calmness during acquisition, a parent attended the child inside the magnetically shielded room. For each subject, two 5-min blocks of continuous MEG data were recorded. If the infant/child felt uncomfortable from lying still, or was getting drowsy and needed a break, data collection was stopped.

Two different experimental conditions were intermixed: *rest and prehension*. Under the *rest condition*, the participant remained motionless for about 10 s while the investigator stood in front of him/her at a distance of approximately 40 cm. Infants and children were visually engaged to prevent head motions. During the *prehension condition*, participants were invited (in case of adults and children) or directed (in case of infants) to squeeze a pipette placed at about 5 cm from their right hand. The pipette, small enough to be comfortably held and squeezed by infants and children, was connected to a pressure transducer to record the pressure exerted during prehension. Adults used a similar device with a button trigger. The pressure profile was synchronized with MEG data, hence permitting the identification of the time points at which squeezing started. This was necessary for MEG data post-processing purposes. The shielded room light was kept dim during all acquisition sessions to minimize distractions. The experimental sessions were recorded with a video camera synchronized with MEG recording to observe behavior and take note of any movement that might have occurred during acquisitions.

### Data acquisition and pre-processing

MEG data were recorded with a sampling rate of 500 Hz. Although data acquisition was halted in cases of significant displacement of the child's head, residual artifacts, including small movements due to chewing/sucking and arm displacements, were rejected during MEG data pre-processing.

**Figure 1:**
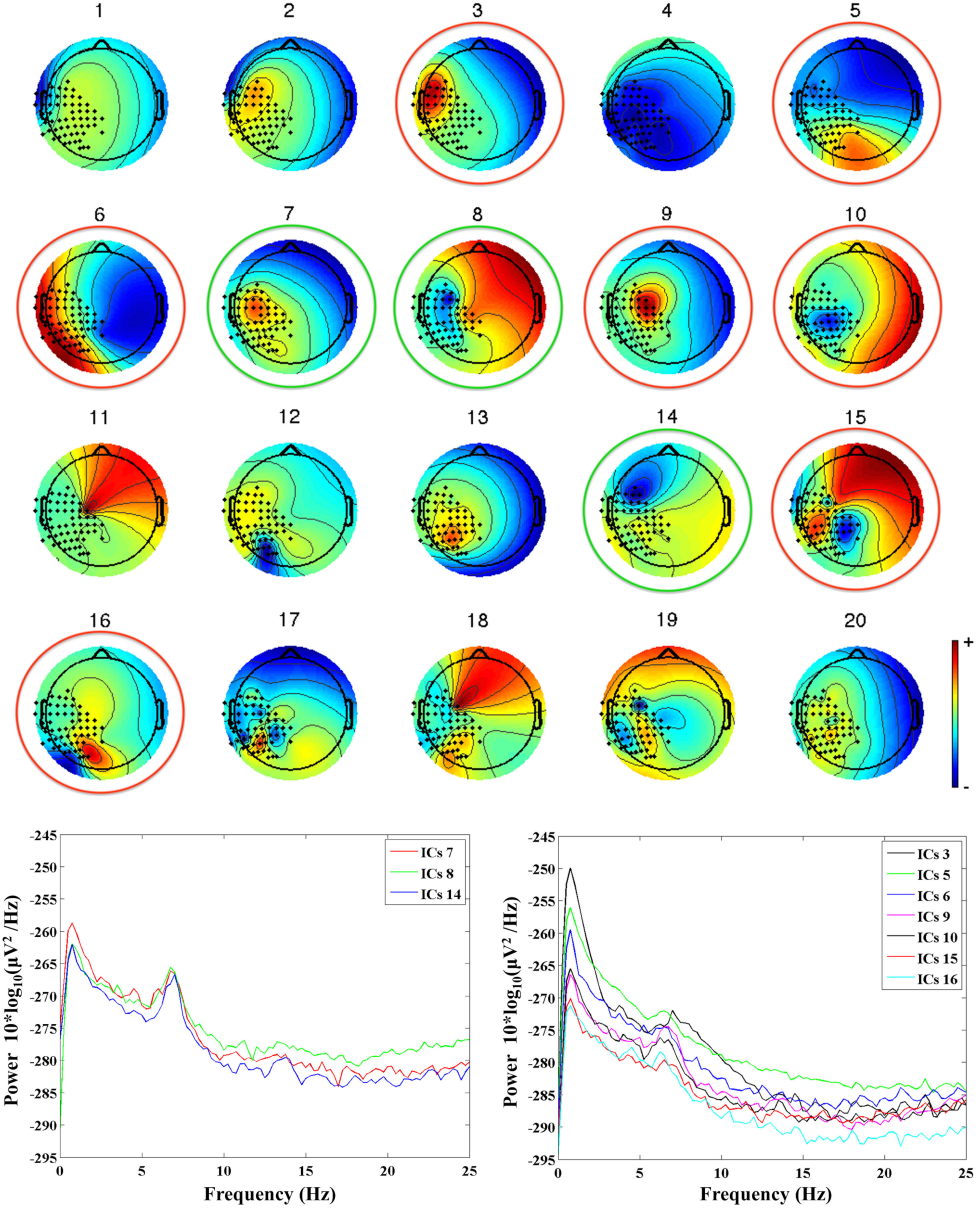
**Upper panel:** the 20 ICs separated from the MEG recordings in an infant at 36 weeks (G2, mu peak frequency at 7.5 Hz). The ICs retained for further analysis are identified by a green circle, whereas the rejected ICs are identified by a red circle. **Lower panels:** power spectra of the retained ICs (on the left hand side), and of the rejected ICs (on the right hand side).

First, MEG channels not working properly were excluded from further analysis. Second, MEG data were band-pass filtered between 0.5 and 40 Hz using a forward-reverse Butterworth filter of the third order. Filtered MEG data were then processed with PCA for dimension reduction, and Independent Component Analysis (ICA, BinICA algorithm with open source toolbox, EEGlab, www.sccn.ucsd.edu/eeglab) was used to separate the independent components (ICs) of interest and to reject those related to artefactual sources. We allowed for a total of 20 ICs, and retained for further processing only those ICs that satisfied two conditions: a topological distribution compatible with the activation of the contralateral sensorimotor cortex, and a clear mu rhythm peak at the individual frequency of the subject, as identified in our previous study (see Berchicci et al., 2011). In general, we retained 2–4 ICs per subject, since the majority of ICs were related to noise, artifacts, MEG channels not working properly, or were seemingly related to brain activity but with a too high content of noise. An example of ICs separated for an infant at 36 weeks is provided in Figure 1, where one can see how ICs with a topological distribution that could be related to the activation of the sensorimotor cortex (e.g., IC6 or IC9), have a frequency content that is mainly due to noise. This type of ICs was not retained to reconstruct the brain signals. The retained ICs (ICs 7, 8, and 14 in the example of Figure 1) were re-projected on the sensors' positions to reconstruct the MEG source signals related to the true brain activity.

### Data analysis

#### Functional connectivity representations

For each subject, the pre-processed MEG signals were segmented according to the 4-s time intervals of rest and prehension identified in our previous study (see Berchicci et al., 2011) for both spontaneous mu rhythm (rest condition) and mu rhythm desynchronization (prehension condition). The rest time intervals were selected during the resting state far from prehension occurrences. The prehension time intervals were selected around prehension (as identified with the pressure transducer profile and the analysis of the video recordings) to include anticipatory cortical activity. Hence, prehension time intervals started 1 s before movement onset.

The patterns of functional dependencies between the pre-processed MEG signals were estimated by means of the Synchronization Likelihood (SL) (data were analyzed using Brainwave v0.9.133.1, http://home.kpn.nl/stam7883/brainwave.html). This measure, introduced by Stam and van Djik (2002), gives a straightforward normalized estimate of the dynamical interdependencies between two or more simultaneously recorded time series. Differently from other connectivity measures such as coherence, this measure, closely related to the concept of generalized mutual information, overcomes the problems related to sub-systems dimensionality, and can be suitable for the analysis of non-stationary data, such as ours. In essence, SL describes how strongly the signal recorded by each channel is synchronized to the signals recorded by the other channels in the array at a given time instant or in a given time span.

For each subject and each experimental conditions (rest and prehension), we calculated one SL matrix for each 4-s time interval within a frequency band centered around the individual mu rhythm peak frequency (IMPF). The IMPF of each subject had been determined in our previous study (see Berchicci et al., 2011), and the frequency band used to calculate the SL matrices had high-pass and low-pass cut-off frequencies at (IMPF − 2 Hz) and (IMPF + 2 Hz) respectively. The SL matrices obtained for all time intervals in each condition were then averaged to obtain one average SL matrix. For each subject we then had two average SL matrices, one for the rest and one for the prehension condition.

To retain only significant functional connections across MEG signals, we thresholded each average SL matrix on the basis of its own SL values distribution, which is expected to be non-Gaussian. We calculated the Median and Median Absolute Deviation (MAD) of the distribution of SL values for each average SL matrix, and defined a new thresholded SL matrix (that we called SL_MAD_ matrix) where only the SL values > (Median + 1 MAD) were retained. All other SL values in the SL_MAD_ matrix were set equal to zero.

Since we analyzed the MEG signals in the sensor space, the SL_MAD_ matrices could still have a bias due to the spatial proximity of adjacent sensors. For this reason, we reconstructed the matrix of Euclidean inter-sensor distances M_Eu_ for all retained MEG channels, and calculated the coefficient of determination *r*^2^ between M_Eu_ and the two SL_MAD_ matrices (one for rest and one for prehension) for each subject. We then retained for further analysis only the subjects for whom both SL_MAD_ matrices satisfied the condition *r*^2^ < 0.1, hence ensuring that the SL values in the SL_MAD_ matrices do not depend on the Euclidean distances across neighboring MEG sensors.

To quantify the overall probability for functional connectivity over the sensorimotor areas at rest and during prehension, for each subject and each experimental condition we calculated the mean SL value in the SL_*MAD*_ matrix as:

(1) $$SL_{MEAN}=\frac{1}{N}\sum_{i,j}SL_{MAD,i,j}$$

where N is the total number of SL values ≠ 0 in the SL_MAD_ matrix.

#### Measures of functional organization

We used graph theoretical concepts to study the topological features of the functional networks represented by the SL_MAD_ matrices. Within this framework, patterns of functional connections are represented as graphs where nodes (in our case the MEG sensors) are linked with edges (if connected). For each subject, we estimated the level of functional organization over the sensorimotor cortex at rest and during prehension by means of segregation and integration measures described below. To this aim, we calculated the segregation and integration measures on the SL_MAD_ matrices, where each SL value (when different from zero) represents the strength of the functional connection (edge) between two given MEG signals (nodes). As the comparison of graphs derived from brain networks requires a step of normalization, for instance by setting a fixed average degree (K) (van Wijk et al., 2010), prior to calculating segregation and integration measures we checked the degrees of the SL_MAD_ matrices of each subject included in the study population, and considered for further processing only those subjects whose SL_MAD_ matrices had the same degree in both rest and prehension (see also sub-Section Statistical Analysis).

***Measures of functional segregation***. Functional segregation in the brain refers to specialized information processing occurring within densely interconnected groups of brain regions (Rubinov and Sporns, 2010). In our case, functional connectivity was calculated by SL_MAD_ matrices in the sensor space, hence segregated neural processing will be suggested by statistical dependencies between clustered channels. We calculated two weighted measures of functional segregation:

1. *Mean clustering coefficient C*. For a given node *i* (in our case, for a given MEG channel *i*), *c*_*i*_ is defined as the fraction of the node's neighbors (other MEG channels) that are directly connected with it (Watts and Strogatz, 1998). At the network level, the *mean clustering coefficient C* reflects the prevalence of clustered connectivity around individual nodes:
  (2) $$C=\frac{1}{n}\sum_{i\;\in \;N}c_{i}=\frac{1}{n}\sum_{i\;\in \;N}\frac{2t_{i}^{w}}{k_{i}\left(k_{i}-1\right)}$$
  where *N* is the set of all nodes in the network, and *n* is the number of nodes, *k*_*i*_ is the degree of node *i* (i.e., the number of links connected to node *i*), and *t*^*w*^_*i*_ is the weighted geometric mean of triangles around *i*. High values of C indicate that a high number of connections exists among neighboring nodes.
2. *Local efficiency E_loc_*. The local efficiency is defined as the average efficiency of the local subgraphs. For weighted connectivity matrices, such as SL_MAD_ matrices, it is:
  (3) $$\text{​}E_{loc}=\frac{1}{2}\sum_{i\;\in \;N}\frac{\sum_{j,h\;\in \;N,j\;\neq \;i}{\left(w_{i,j}w_{i,h}{\left[d_{j,h}^{w}\left(N_{i}\right)\right]}^{-1}\right)}^{1/3}}{k_{i}\left(k_{i}-1\right)}$$
  where, for a given triangle of vertices *i,j,h*, *w*_*i,j*_, and *w*_*i,h*_ are the weights between nodes *i* and *j* and nodes *i* and *h* respectively, and *d*^*w*^_*j,h*_ is the distance between nodes *j* and *h*, i.e., the minimum number of links connecting node *j* to node *h*. *E*_*loc*_ provides an information similar to the mean clustering coefficient C, but adds an indication on how much the system is *fault tolerant*, i.e., how efficient is the communication between the first neighbors of node *i* when it is removed. Local efficiency suggests a connection redundancy that protects the network from local errors and failures (Latora and Marchiori, 2001).

When high functional segregation is found in a functional connectivity matrix, the prevailing functional connections occur across neighboring brain areas (or, as in our case, across signals recorded by neighboring channels).

***Measures of functional integration***. In the brain, functional integration represents the ability to rapidly combine specialized information from distributed brain regions. Although our MEG recordings refer only to the hemisphere contra-lateral to the moving limb, we calculated two measures of functional integration to estimate how easily different brain regions communicate. The measures of functional integration are based on the concept of a path:

1. *Characteristic path length L.* In a graph, the lengths of paths connecting two given nodes estimate the potential for functional integration in the network (Watts and Strogatz, 1998). The *characteristic path length L* is defined as the mean of the distances (i.e., the minimum path lengths) among all nodes pairs:
  (4) $$L=\frac{1}{n}\sum_{i\;\in \;N}\frac{\sum_{j\;\in \;N,j\;\neq \;i}d_{i,j}^{w}}{n-1}$$
  where *d*^*w*^_*i,j*_ is the weighted distance between nodes *i* and *j*, i.e., the minimum number of links connecting node *i* to node *j*. Short paths, corresponding to low values of L, indicate a strong potential for integration within the network.
2. *Global efficiency E_glob_*. In functional connectivity data, such as the SL_MAD_ matrices, paths represent sequences of statistical associations between subsequent pairs of channels. The level of functional integration in the network can be represented by the *global efficiency E*_*glob*_, which is the average inverse shortest path length (Latora and Marchiori, 2001; Achard and Bullmore, 2007). For weighted connectivity matrices:
  (5) $$E_{glob}=\frac{1}{n}\;\sum_{i\;\in \;N}\frac{\sum_{j\;\in \;N,\;j\;\neq \;i}{\left(d_{i,j}^{w}\right)}^{-1}}{n-1}$$
  When high functional integration is found in a functional connectivity matrix, the functional organization of the brain takes advantage of multiple specialized and densely connected areas that are linked with long distance functional connections for a more efficient information processing.

#### Statistical analysis

Linear regression analysis was performed on the individual measures to assess whether any linear correlation exists between age and/or mu rhythm peak frequency and the functional connectivity/efficiency measures.

Groups analysis was performed for each connectivity/efficiency measure on the age groups defined in Section Participants. In each age group, we retained only those subjects for whom the following two conditions were satisfied: (1) the determination coefficient r^2^ between the individual SL_MAD_ matrices at rest and during prehension and the individual M_Eu_ matrix of the euclidean inter-sensor distances was smaller than 0.1, and (2) the degree of the individual SL_MAD_ matrices for rest and prehension was the same. The first condition guarantees that the SL_MAD_ matrices are independent on the inter-sensor distances, and the second condition satisfies the requirement for a reliable comparison of segregation and integration measures. Seven infants (age <6 months) and 4 infants (age <12 months) did not satisfy these conditions and were excluded from further analysis. The characteristics of the age groups are reported in Table 1.

**Table 1:** **Number of subjects included in each age group (N_study_) out of those who met all inclusion and exclusion criteria (N_incl/excl_)**.

**Group**	**N_incl/excl_**	**N_study_**	**Age**		**Mu peak frequency (Hz) (Mean ± SD)**
			**Months**	**Years**	
G1	14	7	2.75–6		4.46 ± 1.12
G2	11	7	6.5–11.75	<1	7.41 ± 0.61
G3	6	6	24–34	2–2.8	8.71 ± 0.64
G4	6	6	36–60	3–5	8.50 ± 0.52
G5	6	6		20–39	10.32 ± 1.20

For each age group, the age range and the mu rhythm peak frequency (Mean ± SD) are also reported.

Statistical analysis was performed using an ANOVA 5 (age groups) × 2 (conditions: rest, prehension) for each dependent variable, i.e., for the individual measure of functional connectivity (*SL*_*MEAN*_), and for functional segregation (*C* and *E*_*loc*_), and integration (*L* and *E*_*glob*_) measures. Post-hoc comparisons were performed using Bonferroni corrections. Statistical significance was set at *p* < 0.05.

## Results

Linear regression analysis on the individual functional connectivity/efficiency measures did not show any significant linear dependence on age or mu rhythm peak frequency. In particular, the coefficient of determination r^2^ between age and *SL*_*MEAN*_, *C*, *E*_*loc*_, *L*, and *E*_*glob*_ ranged between 0.197 and 0.465, and r^2^ between mu rhythm peak frequency and *SL*_*MEAN*_, *C*, *E*_*loc*_, *L*, and *E*_*glob*_ ranged between 0.008 and 0.269, indicating no significant linear correlation between any connectivity/efficiency measure and age or mu rhythm peak frequency.

### Functional connectivity representations

**Figure 2:**
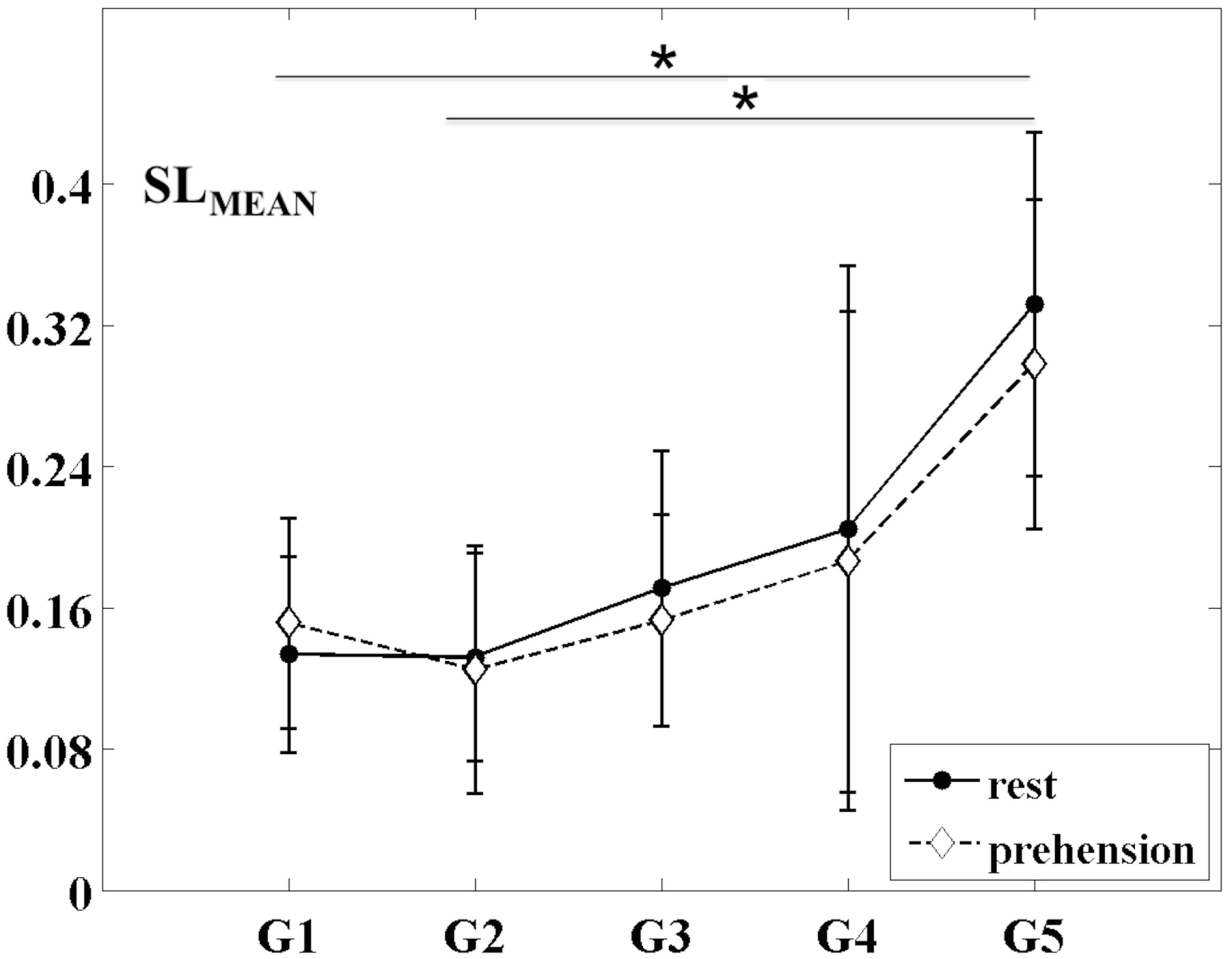
**Group-averaged SL_MEAN_ values (mean ± SD) for the five age groups (G1–G5) and the two experimental conditions (rest and prehension).** ^*^p < 0.05.

The changes of the group-averaged SL_MEAN_ values with age in the two experimental conditions (rest and prehension) are summarized in Figure 2. ANOVA results on the individual measures of SL_MEAN_ showed significant differences across age groups [*F*_(4, 27)_ = 4.546 *p* = 0.006, η^2^_*p*_ = 0.402, power = 0.897], no significant differences between conditions, and no interaction between groups and conditions. During the first year of life (groups G1 and G2), the average SL_MEAN_ values do not differ significantly, whereas around 2 years of age (group G3), SL_MEAN_ values start to increase toward the adult values (group G5). *Post-hoc* analysis showed significant differences only between the group of adults G5 (0.315 ± 0.093) and the groups of infants G1 (0.143 ± 0.056; *p* = 0.015) and G2 (0.129 ± 0.062; *p* = 0.007).

**Figure 3:**
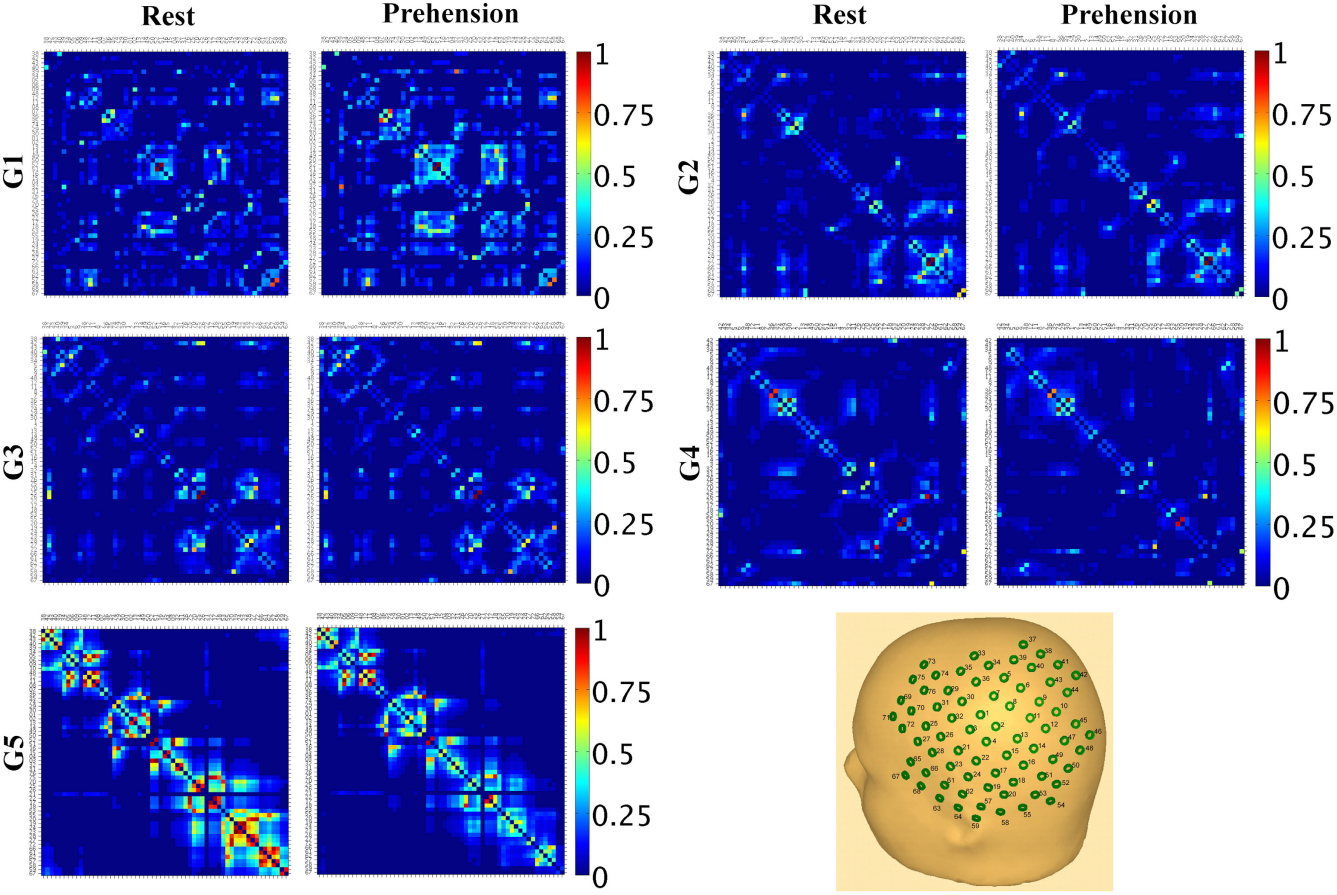
**Examples of SL_MAD_ matrices at rest and during prehension (one example per age group).** G1: infant, 12 weeks; G2: infant, 36 weeks; G3: child, 34 months; G4: child, 41 months; G5: adult, 28 years. A schematic representation of the sensor array and its approximate placement over the left hemisphere is shown in the lower right hand panel. In the SL_MAD_ matrices, the MEG channels are displayed from the medial to the lateral areas.

These results indicate that, during infancy, the likelihood for functional connections over the sensorimotor cortex is much lower than in adulthood, and that it increases with age. Although no statistically significant difference was observed between conditions, it is interesting to observe that, in our group of very young infants (G1), the average SL_MEAN_ is slightly higher during prehension than at rest, whereas in the groups of children and in the group of adults the average SL_MEAN_ slightly decreases during prehension. This fact could deserve further investigation, possibly in larger populations of infants and children. Examples of SL_MAD_ matrices at rest and during prehension for each age group are provided in Figure 3. A typical layout of the MEG sensor array over the head is also shown. However, please consider that the same channels in different SL_MAD_ matrices could be positioned over different brain regions, as no information on the relative positions of the MEG channels with respect to the subject's head could be collected.

### Measures of functional segregation

**Figure 4:**
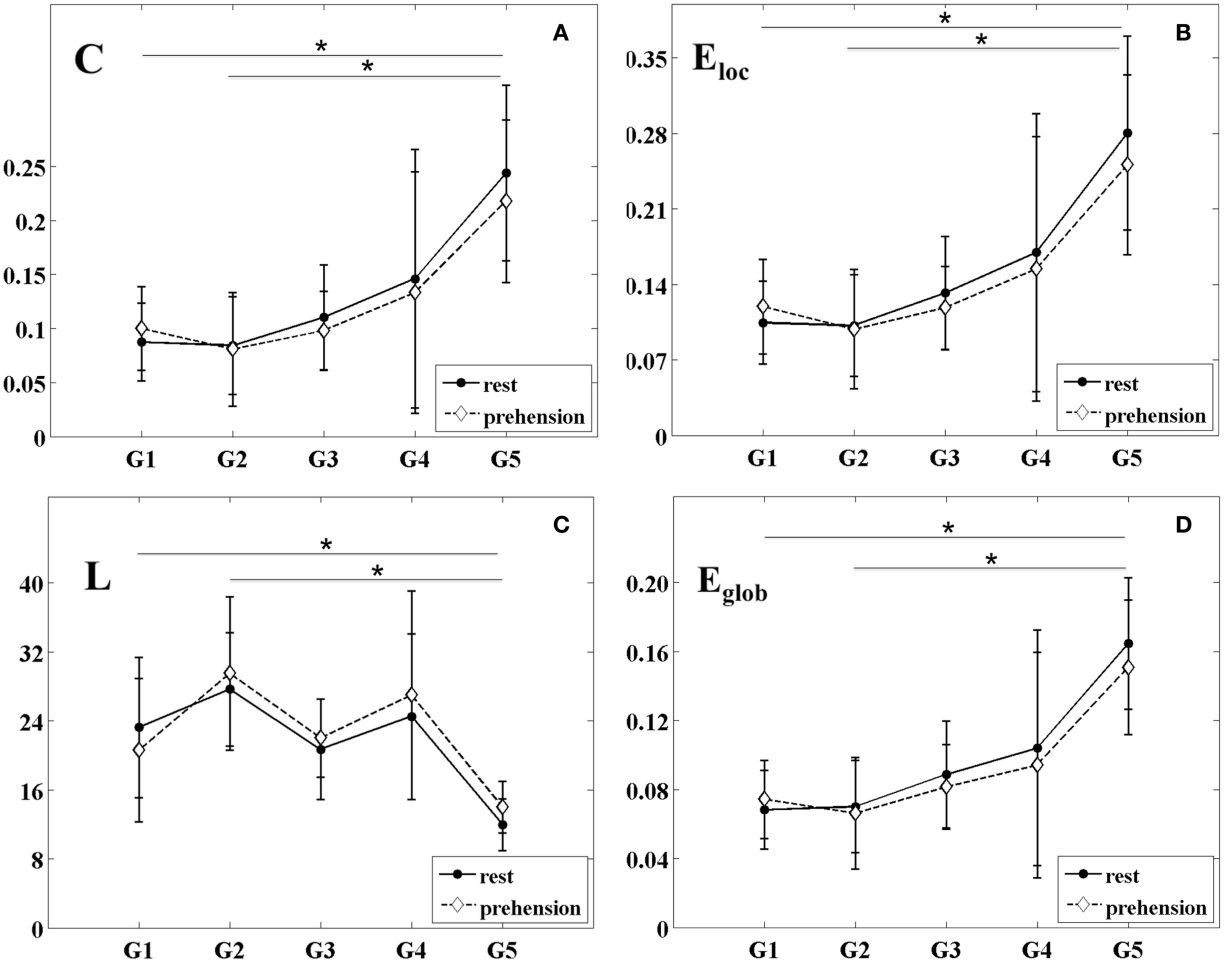
**Group-averaged values (mean ± SD) of the segregation and integration measures for the five age groups (G1–G5) and the two experimental conditions (rest and prehension). (A)** mean clustering coefficient C; **(B)** local efficiency E_loc_; **(C)** characteristic path length L; **(D)** global efficiency E_glob_. ^*^p < 0.05.

The changes of the group-averaged *C* and *E*_*loc*_ values with age in the two experimental conditions (rest and prehension) are summarized in Figures 4A,B. ANOVA results on the individual measures of *C* showed significant differences across age groups [*F*_(4, 27)_ = 4.975 *p* = 0.004, η^2^_*p*_ = 0.424, power = 0.924], no significant differences between conditions, and no interaction between groups and conditions. During the first year of life (groups G1 and G2), *C* remains almost unchanged, whereas around 2 years of age (group G3) it starts to increase toward the adult values (group G5). *Post-hoc* analysis showed significant differences between the group of adults G5 (0.231 ± 0.076) and the groups of infants G1 (0.094 ± 0.037; *p* = 0.011), G2 (0.083 ± 0.047; *p* = 0.005). A tendency toward significant difference was also observed between G5 and G3 (0.104 ± 0.041; *p* = 0.030).

Similarly, ANOVA results on the individual measures of *E*_*loc*_ showed significant differences across age groups [*F*_(4, 27)_ = 5.223 *p* = 0.003, η^2^_*p*_ = 0.436, power = 0.937], no significant differences between conditions, and no interaction between groups and conditions. During the first year of life (groups G1 and G2), *E*_*loc*_ remains almost unchanged, and around 2 years of age (group G3) it starts to increase toward the adult values (group G5). *Post-hoc* analysis showed significant differences between the group of adults G5 (0.266 ± 0.084) and the groups of infants G1 (0.112 ± 0.041; *p* = 0.008), G2 (0.100 ± 0.049; *p* = 0.004), whereas a tendency toward significant difference was observed between G5 and G3 (0.125 ± 0.045; *p* = 0.026).

These results indicate that, during infancy, global measures of functional segregation over the sensorimotor cortex are significantly lower than in adulthood, and that they increase with age. Although no statistically significant difference was observed between conditions, in the very young infants (G1), functional segregation seems to be higher during prehension than at rest, whereas in children and in adults it seems to decrease from rest to prehension. As for SL_MEAN_, this aspect deserves further investigation in larger populations of infants and children.

### Measures of functional integration

The changes of the group-averaged *L* and *E*_*glob*_ values with age in the two experimental conditions (rest and prehension) are shown in Figures 4C,D. ANOVA results on the individual measures of *L* showed significant differences across age groups [*F*_(4, 27)_ = 4.245 *p* = 0.009, η^2^_*p*_ = 0.386, power = 0.873], no significant differences between conditions, and no interaction between groups and conditions. *L* tends to decrease with age, although discontinuously. *Post-hoc* analysis showed significant difference between the group of adults G5 (12.975 ± 3.034) and the group of older infants G2 (28.588 ± 7.575; *p* = 0.007), and a trend toward significant difference between G5 and the group of older children G4 (25.739 ± 10.494; *p* = 0.047).

ANOVA results on the individual measures of *E*_*glob*_ showed significant differences across age groups [*F*_(4, 27)_ = 5.594 *p* = 0.002, η^2^_*p*_ = 0.453, power = 0.952], no significant differences between conditions, and no interaction between groups and conditions. We can see that *E*_*glob*_ remains almost unchanged in infancy (groups G1 and G2), and that it increases with age. *Post-hoc* analysis showed significant differences between the group of adults G5 (0.158 ± 0.037) and the groups of infants G1 (0.071 ± 0.022; *p* = 0.004) and G2 (0.068 ± 0.029; *p* = 0.003). A tendency toward significant difference was observed between G5 and G3 (0.085 ± 0.027; *p* = 0.030).

Both measures indicate a significant difference of functional integration over the sensorimotor cortex between infancy and adulthood, and that functional integration over the sensorimotor cortex increases with age. Although no statistically difference was observed between conditions, both *L* and *E*_*glob*_ seem to indicate a higher functional integration during prehension in the very young infants (G1), whereas in children and in adults functional integration seems to decrease from rest to prehension. As for the other connectivity and segregation measures, this aspect deserves further investigation in larger populations of infants and children.

## Discussion

Our goals were to contribute to the identification of the developmental trajectories of the functional properties over the sensorimotor cortex, and to explore whether any differences could be observed between the rest condition and the execution of a prehension task. To ensure that all findings were related to the sensorimotor cortex, all analyses were performed within a frequency band centered on the individual mu rhythm peak frequency of each subject, as defined in our previous study (Berchicci et al., 2011), since the mu rhythm is known to be the idling rhythm of the sensorimotor cortex. Furthermore, the use of a prehension task for the active condition permitted us to investigate the contribution of sensorimotor processing to the generation of perception and action coupling in infants.

We first observed that the probability of functional connectivity across the sensorimotor areas, estimated by means of *SL*_*MEAN*_, remained almost unchanged during the first year of life, whereas it afterwards increased across groups to reach adult values, significantly different from the infant ones. This increase, occurring after 12 months of age, is in agreement with the suggested pattern of brain maturation in which areas with fronto-temporal connections develop more slowly than other regions (Lebel et al., 2008), and with the notion that motor experience increases the capacity of the whole network to work in an integrated way (Fair et al., 2009; Giedd et al., 2009; Boersma et al., 2011). Unfortunately, no information on the relative position of the MEG sensor array and the subject's head could be collected, therefore we could not precisely ascribe the observed functional connections across MEG signals to specific brain areas. Consequently, our observations on the probability of functional connectivity should be interpreted as global measures over the sensorimotor network.

To support the interpretation of these results, we used graph theoretical concepts to examine the changes occurring in the functional organization of the sensorimotor network across ages, and found that all segregation and integration measures showed a trend similar to *SL*_*MEAN*_: during the first year of life these properties remained almost unchanged, whereas they increased significantly across groups afterwards, as assessed by a marked increase of both segregation parameters (*C* and *E*_*loc*_), by a tendency of *L* to decrease, and by a clear increase of *E*_*glob*_ across age groups.

The age-related increase of the functional segregation parameters (*C* and *E*_*loc*_) is compatible with the observation that the widespread brain electrical activity typical of infants (8 months of age) becomes more localized during early childhood (3 years of age) (Bell and Wolfe, 2007). We hypothesize that the increase of functional connections among adjacent cortical areas, which contributes to the functional specialization of the sensorimotor network and to the onset of a connection redundancy that protects the network from local errors and failures (Latora and Marchiori, 2001), is due to brain maturation, a process that involves both physical growth and the intellectual and/or emotional process of development. Also, the increase of local efficiency over the sensorimotor cortex is in line with the findings by Wu et al. (2013), who observed an age-related increase in the local efficiency of the whole functional networks, which may contribute to the development of modular information processing of functional systems.

Although the findings on the functional integration properties over the sensorimotor cortex should be taken with caution because the MEG sensor array covered only one hemisphere, it is interesting to observe that also these properties increased with age after the first year of life, and that the infant values were significantly different from the adult ones. The irregular trend of *L* is also worth noting, as it might be related to the occurrence of growth spurts that coincide with periods of discontinuous development in cognition (van Baal et al., 2001).

Overall, our findings on the functional segregation and integration properties over the sensorimotor cortex support the notion that maturation contributes to both the functional specialization of the sensorimotor network, and the wiring of more efficient long-range connections among different brain circuits. This interpretation is in agreement with the observations by Gao et al. (2011) on the increase of both local and global efficiency at rest from newborns to infants, and with the findings of Damaraju et al. (2014) that the connectivity strength between more distant networks increases with age in infants between 4 and 9 months. Conversely, our results are only partially compatible with the notion that strong local connectivity in the young brain gradually shifts toward stronger long-distance connectivity with maturation (van Baal et al., 2001; Lebel et al., 2008; Fair et al., 2009; Power et al., 2010; Yap et al., 2011).

Our results on the age-related increase of both local and global efficiency are consistent also with the outcome of other studies on the developing brain, which demonstrated a steady increase of global efficiency after 1 year of age (Fransson et al., 2007; Fan et al., 2011), a positive relationship between age and local efficiency throughout the life span (Supekar et al., 2009; Dennis et al., 2013; Wu et al., 2013), and the involvement of both segregation and integration in the development of the adult fronto-parietal network for adaptive online task control (Fair et al., 2007; Sepulcre et al., 2010). Our findings seem to support this interpretation of the functional evolution of the fronto-parietal network, known to host sensorimotor processing (Pineda, 2005). From this perspective, our results seem to fit well also with the suggestion that perceptual and cognitive developments involve the simultaneous segregation and integration of information-processing streams (Bunge and Wright, 2007), which in turn support the learning processing in infants and children (de Klerk et al., 2014).

Some authors have also suggested that the child brain has a small-world organization that combines the high clustering properties of an ordered network and the short path length of a random network (Fair et al., 2009; Supekar et al., 2009; Power et al., 2010; Boersma et al., 2011). Other authors have observed that, in school children, the brain functional networks evolve from more random toward a more ordered configuration (Smit et al., 2010, 2012; Boersma et al., 2011). Although our hemispheric recordings do not allow for general conclusions on the functional organization of the whole brain, nonetheless our results complement the above mentioned findings by suggesting that, during infancy, the functional properties over the sensorimotor network have a more random organization that, after 1 year of age, shifts toward a more efficient small-world configuration characterized by higher local and global efficiency. It is interesting to observe that this change toward a more efficient functional organization over the sensorimotor network continued throughout childhood until adulthood. The shift from a more random to a small-world configuration could be ascribed to brain maturation, which derives from changes at the structural level, with the thickening of long-range connections among distant brain regions starting during the first year of life (Yap et al., 2011) and with the emergence of inter-hemispheric connectivity earlier than longer-range antero-posterior connections (Bell and Fox, 1992), but is also shaped by experience, which can support the capacity of the network to create long-range connections that are functional to goal-directed actions (Wu et al., 2013; Rotem-Kohavi et al., 2014).

Unfortunately, we could not draw any conclusions on the contribution of sensorimotor processing to the generation of perceptual-motor coupling, since we did not find any significant difference in the functional properties over the sensorimotor cortex between rest and prehension. However, it is worth noting that the pattern of functional organization over the sensorimotor cortex seems to change around 1 year of age. All measures seem to suggest that, during infancy, prehension could be characterized, with respect to rest, by an increased probability of functional connectivity and by increased segregation and integration. This pattern seems to reverse after 1 year of age. These observations, if confirmed in future studies on larger populations of infants and children supported by measurements of motor experience, could indicate that, in young infants, perceptual-motor coupling is accompanied by an increased connectivity within the sensorimotor network to compensate for the lack of functional specialization to accomplish the given task (prehension). The evolution of this functional pattern could be due to the developmental timeline of the infant grasping or to the tight correlation between grasping recognition and execution, which are poor until 6 months of age (Del Giudice et al., 2009), hence requiring the recruitment of more functional resources in early infancy with respect to later ages. On the other hand, the pattern reversal that seems to occur after 1 year of age might indicate that maturation had fostered a more efficient execution of goal-directed actions, in line with the experience-dependent position on perceptual-motor coupling development of the sensorimotor cortex (Heyes, 2001; van Elk et al., 2008; Del Giudice et al., 2009; Cook et al., 2014), and with the concept that sensorimotor experience contributes to the development of more efficient functional networks to support sensorimotor development (Fransson et al., 2011).

We are aware that the impact of our study is limited by a number of factors. First, the shape and size of the MEG sensor array allowed the recording of brain activity originating only from one hemisphere. Consequently, our observations on the small-world topology of the child brain cannot be referred to the whole brain, and the interpretation of the integration measures needs to be cautious. Second, we employed a cross-sectional design for this study. Given that, during infancy and childhood, the brain undergoes a remarkable development at both structural and functional levels, the recorded brain activity could be influenced by the size of the scalp at different ages, which fits differently on the headrest covering the sensor array. Third, the measures of the topological features of the developing sensorimotor network were calculated in the sensor space rather than in the source space, because anatomical information on the baby's head and on its position with respect to the MEG sensor array could not be collected. This condition limits the interpretation of our results in terms of specific cortical areas. Fourth, we are aware of the small sample size of our age groups. However, study protocols like ours are difficult to perform in children and even more so in infants, and the analyses performed are extremely sensitive to noise and to other signal features that further reduced the number of retained subjects. Nonetheless, several research studies in adults and children have been considered reliable even when based on a small number of subjects (Nishitani and Hari, 2000; Simoes et al., 2004; Lepage and Theoret, 2006; van Schie et al., 2008).

In light of these limitations, further work is needed to validate our findings. However, we believe that our results add valuable information to the current knowledge on the functional development of the sensorimotor network during infancy and childhood. The human brain performs its sensory, cognitive and motor functions by dynamically employing highly complex and interwoven neuronal networks, with the first year of life being the most dynamic period of human postnatal brain development. Better understanding of the functional development of these networks during infancy may bring new insights on the pathophysiological mechanisms of neurological development, such as Down syndrome, autism spectrum disorders and cerebral palsy. Further studies employing whole head neuroimaging techniques during motor task execution and complemented with motor experience measurements are therefore needed to support the present findings, and to improve our understanding of the functional development of the sensorimotor network and its contribution to the generation of perceptual-motor coupling in infants.

## Author contributions

The study presented here has been conceived and designed by Marika Berchicci and Silvia Comani. The experiments were performed by Marika Berchicci. Data were analyzed by Gabriella Tamburro, Silvia Comani, and Marika Berchicci. Data have been interpreted by Marika Berchicci, Gabriella Tamburro, and Silvia Comani. The manuscript was written, revised and approved by Marika Berchicci, Gabriella Tamburro, and Silvia Comani.

### Conflict of interest statement

The authors declare that the research was conducted in the absence of any commercial or financial relationships that could be construed as a potential conflict of interest.

## Abbreviations

SL_MEAN_: mean synchronization likelihood
C: mean clustering coefficient
E_loc_: local efficiency
L: characteristic path length
E_glob_: global efficiency.
